# Critical care pharmacy workforce: a 2020 re-evaluation of the UK deployment and characteristics

**DOI:** 10.1186/s12960-023-00810-y

**Published:** 2023-03-31

**Authors:** Mark Borthwick, Greg Barton, Christopher P. Ioannides, Ruth Forrest, Emma Graham-Clarke, Fraser Hanks, Christie James, David Kean, David Sapsford, Alan Timmins, Mark Tomlin, John Warburton, Richard S. Bourne

**Affiliations:** 1 Departments of Pharmacy and Critical Care, Oxford University Hospitals NHS Foundation Trust, Oxford, England United Kingdom; 2 Pharmacy Department, St Helens and Knowsley Teaching Hospitals NHS Trust, England Prescot, United Kingdom; 3 Pharmacy Department, Sheffield Teaching Hospitals NHS Foundation Trust, Sheffield, England United Kingdom; 4 Departments of Pharmacy and Critical Care, NHS Greater Glasgow and Clyde, Glasgow, Scotland United Kingdom; 5 Department of Anaesthetics, Sandwell and West, Birmingham Hospitals NHS Trust, Birmingham, England United Kingdom; 6 Pharmacy Department, Guy’s and St Thomas’ NHS Foundation Trust, London, England United Kingdom; 7 Pharmacy Department, Aneurin Bevan University Health Board, Cwmbran, Wales United Kingdom; 8 Pharmacy Department, Belfast Health and Social Care Trust, Belfast, Northern Ireland United Kingdom; 9 Pharmacy Department, Cambridge University Hospitals NHS Foundation Trust, Cambridge, England United Kingdom; 10 Pharmacy Department, NHS Fife, Kirkcaldy, Scotland United Kingdom; 11 Pharmacy Department, University Hospital Southampton NHS Foundation Trust, Southampton, England United Kingdom; 12 Pharmacy Department, University Hospitals Bristol and Weston NHS Foundation Trust, Bristol, England United Kingdom

**Keywords:** Pharmacy, Hospital, Intensive care, Organisation, Planning, Census, Distribution

## Abstract

**Introduction:**

Critical care pharmacists improve the quality and efficiency of medication therapy whilst reducing treatment costs where they are available. UK critical care pharmacist deployment was described in 2015, highlighting a deficit in numbers, experience level, and critical care access to pharmacy services over the 7-day week. Since then, national workforce standards have been emphasised, quality indicators published, and service commissioning documents produced, reinforced by care quality assessments. Whether these initiatives have resulted in further development of the UK critical care pharmacy workforce is unknown. This evaluation provides a 2020 status update.

**Methods:**

The 2015 electronic data entry tool was updated and circulated for completion by UK critical care pharmacists. The tool captured workforce data disposition as it was just prior to the COVID-19 pandemic, at critical care unit level.

**Main findings:**

Data were received for 334 critical care units from 203 organisations (96% of UK critical care units). Overall, 98.2% of UK critical care units had specific clinical pharmacist time dedicated to the unit. The median weekday pharmacist input to each level 3 equivalent bed was 0.066 (0.043–0.088) whole time equivalents, a significant increase from the median position in 2015 (+ 0.021, *p* < 0.0001). Despite this progress, pharmacist availability remains below national minimum standards (0.1/level 3 equivalent bed). Most units (71.9%) had access to prescribing pharmacists. Geographical variation in pharmacist staffing levels were evident, and weekend services remain extremely limited.

**Conclusions:**

Availability of clinical pharmacists in UK adult critical care units is improving. However, national standards are not routinely met despite widely publicised quality indicators, commissioning specifications, and assessments. Additional measures are needed to address persistent deficits and realise gains in organisational and patient-level outcomes. These measures must include promotion of cross-professional collaborative working, adjusted funding models, and a nationally recognised training pathway for critical care pharmacists.

## Introduction

Care of the critically ill patient is complex, involving multiple interventions provided in a dynamic environment by many healthcare professionals and teams. The most common intervention in healthcare is medication [1]. The complexity of medication safety in the intensive care unit has been clearly outlined and preventative strategies identified [2]. Clinical pharmacists contribute to improved patient safety, but also to better patient outcomes, particularly when working directly within the multiprofessional critical care team [3]. However, the impact of clinical pharmacist medicines optimisation activity is affected by pharmacist staff resources (number and level of practice) [4].

The need for specialist clinical pharmacists in critical care (critical care pharmacists) is well recognised internationally [5, 6]. In the UK, standards on critical care pharmacist staffing ratios and level of practice are in place to support practice delivery [7, 8]. However, there remains challenges to national delivery of these standards, despite efforts to develop advanced-level practice training programmes [9], scope of practice (e.g., independent prescribing) [10], and credentialing [11]. Most recently, the COVID-19 pandemic challenge clearly exposed the limitations of critical care workforce capacity, including for pharmacists [12].

We have previously described the United Kingdom (UK) critical care pharmacy workforce deployment and characteristics in 2015 [13]. Geographical variations were identified in staffing numbers, level and scope of practice, as was a notable lack of weekend services provision [13]. In 2015, we reported that nearly all UK critical care units (98.6%) had a designated clinical pharmacist [13], with comparable results obtained from the 2016 non-medical workforce survey in England, Wales and Northern Ireland [14]. Both reports identified that fewer than half of pharmacists (47%, 87/186) worked at advanced-level practice (Advanced Stage II) at the time [13, 14].

The aim of this evaluation was to provide a workforce status update for 2020 to understand and compare UK critical care pharmacy staffing developments since the 2015 census.

## Methods

### Survey registration

The study was classified and registered as service evaluation at the Oxford University Hospitals NHS Foundation Trust (Ref 6954).

### Survey design

The questionnaire design was based on the previous United Kingdom (UK) adult critical care pharmacy workforce survey conducted in 2015 [13]. The major difference between the 2020 and 2015 questionnaires was that we specifically sought data at individual critical care unit level rather than at an organisational level. This allowed for greater workforce detail, whilst still enabling direct comparison with the 2015 results [13]. For each critical care unit, we sought data on the level and number of adult critical care beds [15] and the demographics of the assigned clinical pharmacy services [15]. Anonymised data were gathered for each clinical pharmacy professional’s time dedicated to critical care services with detailed breakdown on service allocation (e.g., independent patient review; multiprofessional ward rounds), as well as details of service provision over a 7-day week, and systems of service continuity. One whole time equivalent (1.0 wte) was defined as 37.5 h per week. A level 3 bed (intensive care bed) was treated as equivalent to two level 2 beds (high dependency care beds). Critical care beds were defined as level three or level two beds.

Clinical pharmacist demographics required assignment to a level of practice according to the Advanced Pharmacy Framework (APF) of the Faculty of the Royal Pharmaceutical Society (RPS) [16], where level of practice is clearly described in a progression from Advanced Stage I (ASi), to Advanced Stage II (ASii), and Consultant-level (Mastery) in adult critical care pharmacy [16]. The APF comprises 34 competencies across 6 clusters (expert professional practice, collaborative working relationships, leadership, management, education training and development, and research and evaluation) [16]. The questionnaire was created in an electronic format (SurveyMonkey Inc, Palo Alto, California, USA www.surveymonkey.com) to allow for ease of dissemination. Prior to distribution, the questionnaire was piloted on 5 UK critical care units in May 2020. No significant changes were required, pilot data were removed to ensure the database was blank prior to distribution of the final questionnaire.

### Survey dissemination and response follow-up

We created a master list of adult critical care units from the Intensive Care National Audit and Research Collaboration case-mix programme (ICNARC CMP) [17], for critical care units in England, Wales and Northern Ireland. Critical care units in Scotland were identified from the Scottish Intensive Care Society Audit Group (SICSAG) [18]. Inclusion criteria were all adult critical care units within the United Kingdom, excluding obstetric high dependency units. The master list detailed 351 registered critical care units and was used to monitor the extent of the questionnaire response.

The questionnaire was distributed electronically on 10th June 2020, via the United Kingdom Clinical Pharmacy Association (UKCPA) Critical Care Group message board. A convenience sampling technique was employed; participation was on a voluntary basis with no financial incentives. Although most UK critical care pharmacists (CCPs) are members of the UKCPA [19], we asked recipients to forward the questionnaire and link to relevant local networks and contacts for completion. Furthermore, we identified regional CCP leads to co-ordinate and promote responses. We provided weekly feedback on response rates overall and regionally via the UKCPA Critical Care Group message board for 4 weeks. Following this, regional leads contacted individual CCPs or pharmacy clinical services manager of non-responding units to encourage completion. Continued non-response was followed by direct telephone contact with the critical care unit. Participants were specifically directed to answer questions as they pertained to the period just before the first COVID-19 pandemic surge impacted health services (i.e. up to January 2020). The questionnaire closed on 12th November 2020.

### Data analysis

Survey data were managed and analysed in Excel (Microsoft Excel 2016, Redmond USA). A statistics add-in [Real Statistics Resource Pack Software (Release 4.3) Copyright (2013–2015) Charles Zaiontz www.real-statistics.com)] was utilised for statistical analysis. A *p* value < 0.05 was considered statistically significant. Data were assessed for normality (Shapiro–Wilk test) and as they were non-parametric, the Kruskal–Wallis test was used to analyse group variance, and Mann–Whitney *U* test used for pairwise analysis.

## Results

### Survey response

A total of 341 responses were received, containing data for 337 of the 351 Intensive Care National Audit and Research Centre (ICNARC) Case-Mix Programme (CMP) / Scottish Intensive Care Society Audit Group (SICSAG) locations (96.0%), and an additional 4 unregistered locations. Three locations had no critical care beds and 7 locations were reported as closed. Data for 5 locations had been aggregated into other entries, and data for 3 locations had been split out into separate, additional responses.

Overall, there were 329 submissions containing data for 334 critical care units (England 250, Scotland 58, Wales 16, Northern Ireland 10). These units were located in 203 organisations (England 157, Scotland 23, Wales 14, Northern Ireland 9).

### Pharmacist staffing availability

Overall, 328/334 (98.2%) critical care units had specific pharmacist time dedicated to the unit. Each week, there were 234 wte pharmacists working on critical care Monday to Friday (8776.4 working hours), with an additional 6.4 wte pharmacists (241.4 working hours) on Saturdays and Sundays (2.7% of total working time spent on weekends).

**Fig. 1:**
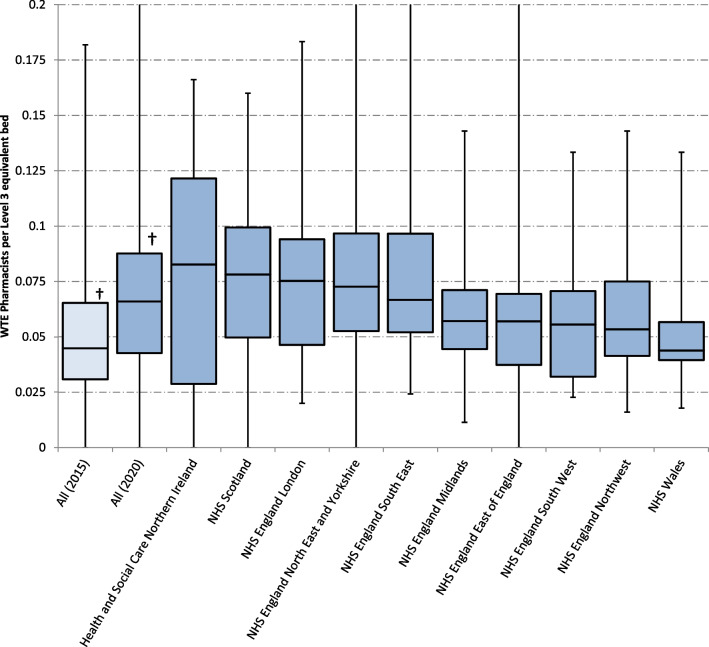
Whole time equivalent pharmacists per Level 3 equivalent bed, All 2015 versus All 2020, and displayed by NHS Region in 2020. ^†^Median difference + 0.021 wte per Level 3 equivalent bed, Mann–Whitney *U*, *p* < 0.0001

Box and whisker plots of whole time equivalent pharmacist time per Level 3 equivalent bed (Monday to Friday) are shown in Fig. 1. As an overall figure and by UK National Health Service (NHS) region. Significant inter-regional differences exist (Kruskal–Wallis, *p* = 0.009).

The overall median weekday pharmacist input to each level 3 equivalent bed was 0.066 (0.043–0.088) wte. There is greater input across the UK in 2020 compared with 2015 (Fig. 1).

The required staffing level for the beds identified in the survey, the expected continuity figure, actual provision, and the gap for a Monday to Friday service are shown (Table 1).

**Table 1:** Critical care Level 3 equivalent beds and weekday pharmacist staffing level (required by standards and actual) by country

	Level 3 equivalent beds	Required wte (Mon–Fri)	Required Continuity	Total Required wte (Mon–Fri)	Actual wte (Mon–Fri)	Gap (% of actual wte)
England	3034	303.4	60.7	364.1	195.7	168.4 (86.1)
Scotland	365	36.5	7.3	43.8	25.1	18.7 (74.5)
Wales	154.5	15.5	3.1	18.6	7.1	11.5 (162.0)
Northern Ireland	85	8.5	1.7	10.2	6.1	4.1 (67.2)
Total	3638.5	363.9	72.8	436.7	234	202.7 (86.6)

For a 7-day service the required provision is 509.4 wte, plus 101.9 wte for continuity. The actual provision is 240.5 wte, yielding a gap of 370.8 wte pharmacists.

### Pharmacist level of practice

**Fig. 2:**
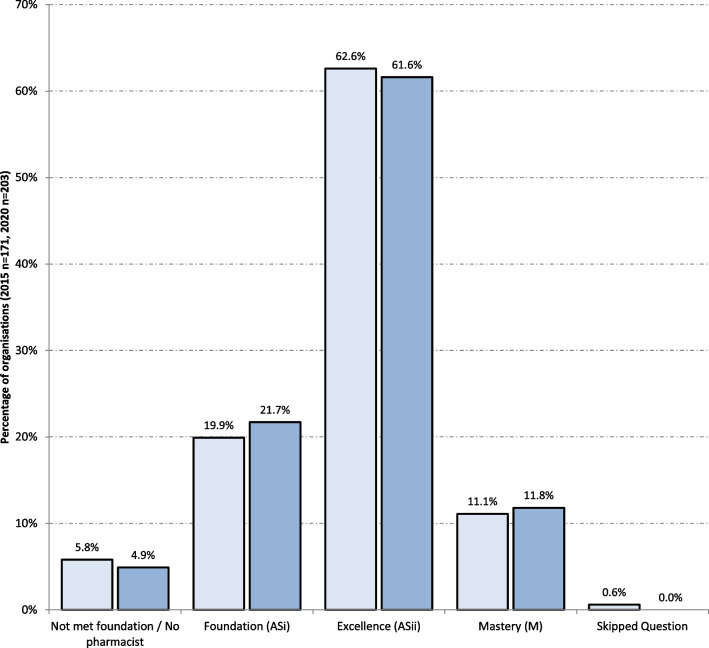
Highest competence level of critical care pharmacy practice expertise available to organisation (2015 light blue bars, 2020 dark blue bars)

The highest competence level of the critical care pharmacist competence available within an organisation is shown in Fig. 2.

### Pharmacist continuity of service provision

**Fig. 3:**
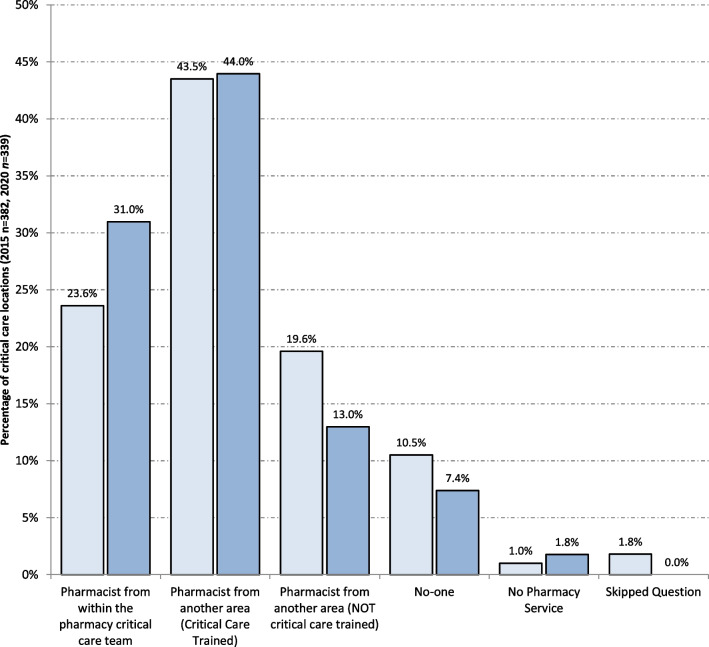
Characteristics of continuity of service arrangements for critical care pharmacists (2015 light blue bars, 2020 dark blue bars)

Three-quarters of all units reported that continuity of pharmacist service provision was provided by pharmacists from within the critical care pharmacist team or by pharmacists from another clinical area who are critical care trained (Fig. 3).

### Pharmacist clinical and professional support activity

**Fig. 4:**
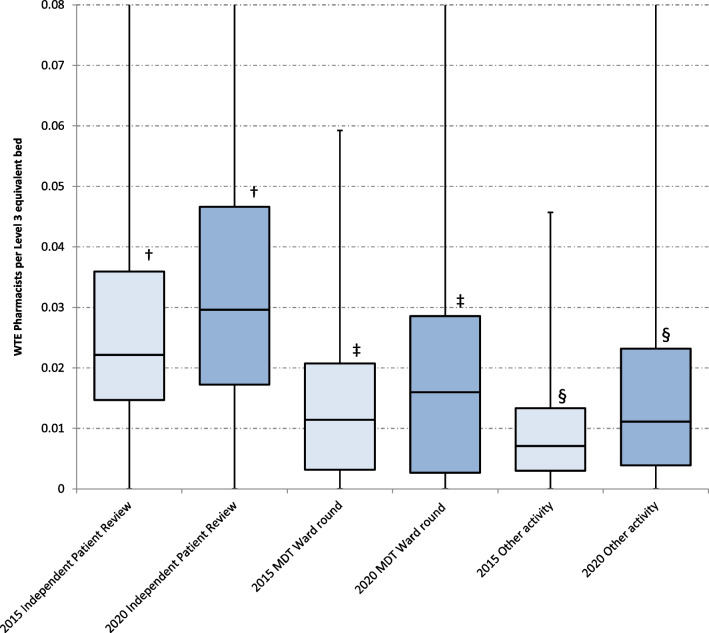
Box and whisker plots of whole time equivalent pharmacist time per level 3 equivalent bed (Monday–Friday) by activity.†median difference + 0.007 wte per level 3 equivalent bed, Mann–Whitney U, *p* < 0.001, ‡ median difference + 0.005 wte per level 3 equivalent bed, Mann–Whitney U, *p* < 0.001, § median difference + 0.004 wte per level 3 equivalent bed, Mann–Whitney U, *p* < 0.001

Each week, pharmacists spend 27.9% of critical care time attending the multiprofessional (MDT) ward round, 46.7% of time on independent patient review and 25.4% of time other professional activities for critical care. A comparison of these pharmacy activities between 2015 and 2020 is shown in Fig. 4. There is greater input across the UK in 2020 compared with 2015 for each activity.

Pharmacists routinely contributed to the multi-disciplinary team (MDT) ward round in 252 critical care units (75.4%). The results of an exploratory analysis comparing pharmacy services where pharmacists attend the MDT ward round for any time, compared to services where the pharmacist never attends the MDT ward round are shown in Table 2.

**Table 2:** Characteristics of critical care units and pharmacy teams of services by pharmacist MDT ward round attendance

	Any MDT round input (n = 252, 75.4%)	No MDT round input (n = 82, 24.6%)
Size (L3 equivalent beds) Median (IQR)	9.5 (7–14.5)	5.5 (4–10)
Highest level: Mastery	10.9%	12.2%
Highest level: ASii	61.9%	34.1%
Highest level: ASi	24.3%	39.0%
Highest level: Not met	2.8%	14.6%
Total wte/L3 bed equivalent. Median (IQR)	0.070 (0.050–0.089)^†^	0.050 (0.027–0.079)^†^
Pharmacist round wte/L3 bed equivalent. Median (IQR)	0.027 (0.015–0.043)^†^	0.040 (0.023–0.067)^†^

†Mann–Whitney *U*, *p*<0.001)

Some 71.9% (240/334) of critical care units had prescribing pharmacists as part of the team. These pharmacists were available for 6080 h per week (67.4% of pharmacist’s hours).

### Pharmacist service funding

Pharmacists were funded by the pharmacy department in 233 units (69.8%), by critical care in 25 units (7.5%), or by both pharmacy and critical care in 47 units (14.1%). In 2 units (0.6%) there was an alternative funding stream, in 6 units (1.8%) there is no pharmacy service and for 21 units (6.3%) the funding source was not known to the respondent.

### Other resources

Pharmacy technicians and assistants provided a total of 25.1 wte to 85 critical care units (25.4%) (Monday to Friday).

Electronic prescribing was recorded as being used in 156 critical care units (46.7%), (England 128/250, Scotland 19/58, Wales 0/16, Northern Ireland 9/10).

## Discussion

### Main results

This comprehensive picture of UK critical care pharmacy workforce shows that the quantity of pharmacy provision has significantly increased since 2015 [13]. Progress has been made on time spent across all three broad categories of activity—independent patient review, multiprofessional ward round participation, and professional support activities (e.g., clinical governance, teaching, service improvement). Almost three-quarters of critical care units now have access to clinical pharmacists with the required minimum level of critical care competence, and a high proportion of critical care pharmacists are prescribers.

Continuity of care arrangements have marginally improved since 2015, with fewer untrained pharmacists being utilised, and fewer units reporting no cover is provided. The highest available competence level in the organisation is very similar to the position in 2015. However, most critical care pharmacy services still fall below minimum standards for weekday services, and disappointing progress made with respect to weekend services.

These results are underpinned by an excellent response rate of 96%, with data now captured to the level of ‘critical care unit’, rather than per organisation. Self-reporting of pharmacist competence level remains a limitation, although consistency in methodology enables direct comparison with historical workforce data [13].

These UK critical care pharmacist workforce results display some commonality with workforce data from the United States of America (USA) [20, 21]. In Newsome’s USA report [20], the majority of participants (76%) expressed a need for additional critical care pharmacists at their institutions. UK data compares favourably with US workforce data with nearly all UK units having clinical pharmacist services, compared to approximately 70% in USA ICUs [21]. Direct comparison with Canadian critical care pharmacist availability data was not possible due to differences in response rate and survey questions [22].

Greater awareness of national standards [7], the introduction of an NHS England commissioning document for critical care services [8], and NHS Scotland standards [23], in a pre-pandemic landscape, may have contributed to a climate of investment in critical care pharmacists. These standards are based on an appreciation of the benefits for patient outcomes that come from improvements in the quality and medication safety clinical pharmacists bring to critical care multiprofessional team working [3]. Nevertheless, for these medicines optimisation roles to be delivered, the required resources need to be in place. Poorly resourced units limit activity to identifying medication errors [4]. Similarly, in the USA workforce survey [20], clinical pharmacists perceived that higher patient: pharmacist ratios led to unsafe patient care, and clinical pharmacist understaffing may be a factor in burnout [24]. Moreover, lack of service continuity for periods of leave and weekends may be a stressor, particularly to pharmacists in critical care [25]. More understanding of burnout syndrome risks and the impact on workforce recruitment and retention is required for UK clinical pharmacy professionals.

The increased activity by critical care pharmacists in attending the multiprofessional ward round is welcomed. The ward round facilitates contributions of pharmacists to patients care, e.g., reducing patient adverse drug events [26]. Multiprofessional ward rounds have benefits for team working and co-ordination [27] that are associated with improved patient outcomes [28]. Multiprofessional rounds support effective team working, dependability and task allocation, emphasising that single professions and roles in critical care areas should not be considered in isolation and appropriate co-ordination of tasks is required in such a high-intensity, clinically unpredictable and acute care area [29].

Progression in the proportion of advanced-level pharmacists (ASii) is a welcome finding. Effective systems in patient safety require not only the availability of key healthcare professionals, but those with the right level of training. The combined availability of pharmacists practising at a higher level contributes to improved medicines optimisation outputs [4, 30]. Similarly, more advanced practice as implied by independent pharmacist prescribing [31], is available on the majority of critical care units. This prescribing role availability compare very favourably with US data [21], and is in line with previous projections for UK critical care prescribing capability [10].

However, some NHS regions have approximately half the median weekday provision of other regions. The overall gap across the UK is 203 wte pharmacists to meet minimum weekday standards. In absolute numbers, this is not a large number of posts, for comparison there are at least 17,615 wte nurses in critical care in England and Wales [32], however this deficit represents 6 posts for every 10 critical care units in the UK.

Only a minority of pharmacist posts are funded by critical care departments, with the majority still funded by pharmacy departments. Such a disparate and uncoordinated funding model makes it difficult to prioritise service resourcing and provision and makes the critical care provision vulnerable to intra-departmental differences in service vision and goals. Pharmacy managers have conflicting priorities related to medicines optimisation dashboards, e.g., requiring a staff deployment focus on clinical areas with high volumes of patient turn-over, to meet basic operational performance indicators such as medicine reconciliation and patient discharges, within limited resource. This is particularly true when tackling weekend services [33].

The lack of progress in weekend service provision remains a concern. In the USA, weekend services were less common than weekday services, although many activities still had much better provision than in the UK [21], with greater than 50% availability in key areas such as evaluating /monitoring drug therapy, pharmacokinetic monitoring, and formal pharmacotherapy consults. Only 2.7% of all UK critical care pharmacists time is deployed at weekends, it is very unlikely that UK pharmacists have sufficient job time available to match the extent of weekend activities reported in the USA. This is despite data demonstrating a significant increase in the rate of critical care pharmacist interventions on a Monday compared to the rest of the week, and a higher rate of contributions on weekends in those services that do maintain a weekend service [30].

Unlike in the USA [34], the UK does not yet have a recognised critical care pharmacist training programme. The need for [35], and format of [9], an advanced-level training programme for critical care pharmacists has been identified, but national delivery and credentialling remains a challenge. Greater organisational workstream alignment between pharmacy bodies and intensive care specialty groups can aid pharmacy service developments. An example from the UK is pharmacist membership of the Faculty of Intensive Care Medicine [36]. This recent development is in recognition of the mutual benefits of closer multiprofessional group working and opportunities such as the acceleration of advanced-level practice for clinical pharmacists in the specialty.

Standards of practice described for UK critical care pharmacists are commensurate with statements in other territories, such as Australia and New Zealand [6], and the USA [37]. Standards in the USA are long established and have recently been updated, although implementation is variable [20]. Standards in Australia [6] are strongly endorsed by the College of Intensive Care Medicine of Australia and New Zealand [38]. The implications for the clinical pharmacy workforce, including the need for training and recognition, are highlighted [39]. To address the workforce challenge, a step-change in critical care pharmacist funding, training and workforce modelling is required at a national level. Indeed, this vision has been captured in the recent recommendation for a national costed model for critical care pharmacists in England, to support investment in critical care pharmacists to benefit services and patient care [40]. Nevertheless, the overall effect of critical care pharmacy workforce shortages, in addition to those of medical and nursing staff, on patient safety and care quality provision was not explicit [40]. This will require the input of national health bodies to co-ordinate a strategy review and action plan, and possibly needs to be driven by the specialty in its wider sense, rather than pharmacy on its own. This national work must include further research into the extent and risk factors for burnout syndrome for clinical pharmacy professionals working in critical care areas.

## Conclusions

This pre-pandemic UK workforce study, with an almost complete response rate, demonstrates that clinical pharmacy input to adult critical care is improving, but that the improvement is less than is needed and highlights large regional variations. The availability of direct pharmacy services to critical care at weekends remains inadequate. Cross-professional collaborative working, appropriate funding models and a nationally recognised training pathway are all required to address the gap.

## Data Availability

Data are available from the corresponding author on request.
